# New Paste Electrode Based on Copper and Gallium Mixed Metal Oxides-Decorated CNT for Highly Electrocatalyzed Hydrogen Evolution Reaction

**DOI:** 10.3390/ijms26189057

**Published:** 2025-09-17

**Authors:** Claudio Barrientos, Silvana Moris, Dana Arias, Gina Pecchi, José Ibarra, Galo Ramírez, Leyla Gidi

**Affiliations:** 1 Centro de Investigación de Estudios Avanzados del Maule (CIEAM), Vicerrectoría de Investigación y Postgrado, Universidad Católica del Maule, Av. San Miguel 3605, Talca 34809112, Chile; cbarrientos@ucm.cl (C.B.); smoris@ucm.cl (S.M.); 2 Physical Chemistry Department, Faculty of Chemical Sciences, University of Concepción, Víctor Lamas 1290, Concepción 4070386, Chile; darias@udec.cl (D.A.); gpecchi@udec.cl (G.P.); 3 Departamento de Química Inorgánica, Facultad de Química, Pontificia Universidad Católica de Chile, Av. Vicuña Mackenna 4860, Santiago 8331150, Chile; jfibarra@uc.cl; 4 Millennium Institute on Green Ammonia as Energy Vector (MIGA), Av. Vicuña Mackenna 4860, Macul, Santiago 7820436, Chile; 5 Departamento de Medicina Traslacional, Facultad de Medicina, Universidad Católica del Maule, Av. San Miguel 3605, Talca 34809112, Chile

**Keywords:** paste electrode, mixed metal oxides, electrocatalyst, water splitting, clean energy

## Abstract

H_2_ has become one of the most attractive alternatives to replace fossil fuels in clean energy production, but large-scale production remains a challenge. A key step toward this goal is to develop new efficient electrocatalysts for H_2_ production. This work presents a new mixed metal oxides-decorated CNT paste electrode (MMO@C), which is highly electrocatalytic, for use in the hydrogen evolution reaction (HER). MMO@C is synthesized by a solvothermal method and used as an easy-to-prepare paste electrode. XPS and X-ray analysis indicate that the electrocatalyst corresponds to a mixed surface of Ga_2_O_3_-CuO-Cu_2_O-Cu(OH)_2_@C. The MMO@C electrocatalyst shows a positive *E_o_* of 0.12 V vs. RHE at −10 mA cm^−2^ towards the HER in a neutral medium. In neutral and alkaline media, the presence of Ga_2_O_3_ facilitates the reduction of CuO to Cu(I) species, which is followed by the formation of Cu(s) active sites. Therefore, the excellent electrocatalytic performance toward the HER in a neutral medium is attributed to the synergistic effect between gallium and copper oxides on the electrode surface. The prominent H_2_ production using MMO@C electrocatalyst is 1.31 × 10^−2^ mol cm^−2^, with a turnover number (TON) of 39,423, a turnover frequency (TOF) of 13,141 h^−1^, and a faradaic efficiency (FE) of 94.3%. Although the Tafel slope reveals slow reaction kinetics, the outstanding onset potential allows for the coupling of the electrocatalyst to renewable energy production systems, making it an attractive candidate for producing green H_2_ and for application in membrane water electrolyzers.

## 1. Introduction

To reduce global greenhouse gas emissions and dependence on fossil fuels, most countries around the world are moving towards developing renewable energy sources [1]. An alternative to fossil fuels is hydrogen, which can be produced by electrolyzing water using electric current to separate it into H_2_ and O_2_. Water electrolysis produces no greenhouse gases, and when the electricity used to power the process comes entirely from renewable sources the resulting source is “green” hydrogen [2]. H_2_ has broad application prospects in the advancement of clean energy due to its extremely high energy density, renewable capacity, and zero carbon content [3]. It is even believed that in the future H_2_ will serve as the main energy storage technology, central heating fuel, and primary transportation fuel for cars, trucks, airplanes, and more [2]. Despite numerous research efforts in the design and development of electrocatalysts, the performance of water electrolysis is still insufficient for commercialization [4]. Ongoing research on innovative materials and understanding of their electrocatalytic mechanisms are critical for designing more efficient and stable electrolyzers [4]. Although platinum, ruthenium, and iridium-based materials have long been considered the reference electrocatalysts for electrochemical water splitting, their application is hampered by their high economic cost and limited abundance [5,6]. Recent research has allowed the effective design of electrocatalytic materials for H_2_ production based on transition metals such as cobalt (Co), nickel (Ni), copper (Cu), and iron (Fe), studied as oxides [7,8,9,10] and hydroxides [11,12,13,14] under different pH conditions. Cu-based electrocatalysts are a promising option for large-scale H_2_ production due to the low cost, high abundance, high electrical and thermal conductivity, and electrocatalytic versatility of Cu [15]. Cu is easily recyclable, reducing the need to mine new metals [16] and displays strong corrosion resistance compared to Co, Ni, and Fe [17]. Some copper oxides are advantageous due to their low preparation costs and diverse oxidation states. In this context, monovalent and divalent copper oxide (Cu_2_O and CuO) are considered excellent choices due to their high stability, enhanced electrochemical activity, and superior redox capabilities compared to other transition metal oxides [9,18,19]. For this reason, they are highly attractive materials to produce H_2_ [20] and for other reactions of energetic and environmental interest. As an example, H. Son et al. proposed a facile oxidation method to fabricate flexible and scalable Cu/Cu_2_O/CuO nanoleaves with a self-initiated charge transport platform via induced oxidation to enhance electrochemical water splitting, with an H_2_ production of 4.76 μmol cm^−2^ [21]. In a study by Y.J. Seo et al., the formation of a Cu_2_O/CuO/Cu(OH)_2_ layer was reported for the photoelectrochemical hydrogen evolution reaction (HER), and it was proposed that the generation of sufficient cation vacancies was closely related to the electronic conductivity and charge transfer performance. The results confirmed the presence of a slightly thin Cu(OH)_2_ film as an efficient cocatalyst, which led to a significant enhancement in the current density [20]. Regarding the use of monovalent and divalent copper-based materials as electrocatalysts for other reactions of energetic and environmental interest, N. Mumtazah et al. reported the effects of copper (I) and (II) oxide electrocatalysts on the oxidation of 5-hydroxymethylfurfural (HMF), exhibiting higher electrocatalytic activity for the dominant Cu_2_O electrode [22]. In another recent research, N. Dong et al. proposed the use of hierarchical Cu/Cu_2_O/CuO nanosheets for the electrocatalytic conversion of nitrate to ammonia. The authors indicated that the electronic interaction and interface effect between Cu/Cu_2_O/CuO allow the regulation of the d-band center of Cu and the control of the adsorption energies of the intermediates [23]. On the other hand, gallium-based materials attract attention due to their interesting properties. With a low melting point of 30 °C and the widest liquid temperature range between 30 °C and 2400 °C, gallium combines easily with other metals or nonmetals to build composite materials [24]. However, research on the construction of gallium-based materials for H_2_ production is relatively scarce [24]. A. Kakoria et al. applied highly porous β-gallium oxide (β-Ga_2_O_3_) nanofibers with a specific surface area of ~100–300 m^2^ g^−1^ as an efficient bifunctional electrocatalyst for H_2_ and O_2_ production. The β-Ga_2_O_3_ nanofibers electrocatalyzed the ORR at an onset potential of 0.84 V (vs RHE), and for the HER, although the onset potential was −0.34 V (vs RHE), the current density was visibly better than the Pt/C catalyst. The attractive performance related to low onset potential and high current density for both reactions was solely attributed to the large surface area and unique morphology presented by the material [25]. Y. Zang et al. showed a synergistic effect between Ga and Co in the preparation of ultrathin Ga-doped CoP nanosheets (Ga-CoP NSs). Ga-CoP NSs deliver improved HER electrocatalytic activities. Density functional theory simulations indicated that the Ga dopant could systematically enhance the HER activity of CoP by improving the H_2_O adsorption, weakening OH adsorption, and optimizing the H adsorption/desorption [26]. Recently, L. Chen et al. developed an innovative and energy-efficient Cu_5_Ga_1_ bimetallic electrocatalyst for CO_2_ conversion, where the HER acts as a competing reaction. In this study, it was determined that Cu_5_Ga_1_ exhibits a much higher activation energy for H_2_ production (39.7 kJ mol^−1^ at −1 V vs. RHE) compared to the reference Cu electrocatalyst (27.3 kJ mol^−1^ at −1 V vs. RHE). The authors observed that the presence of Ga shows a remarkable effect in preventing Cu oxidation under ambient conditions. This effect is probably due to the high oxophilicity and low electronegativity of Ga, which enhances the electrocatalytic properties of the bimetallic system [27]. Therefore, the use of mixed metal oxides of gallium and copper as electrocatalysts for hydrogen evolution seems convenient. In general, metal oxides can further enhance their electrochemical properties when supported on conductive materials, such as multi-walled carbon nanotubes (MWCNTs) [28,29,30]. The highly resonant MWCNTs system and their cylindrical structures enable directed electron flow, improving both redox processes and charge transfer [31]. For these reasons, it is proposed that Ga_2_O_3_ can enhance the electrocatalytic performance of copper species when located in proximity over a MWCNTs surface. This work proposes a new paste electrode composed of MWCNTs decorated with mixed metal oxides of gallium and copper (MMO@C) to electrocatalyze the HER. The novelty of this work lies in the analysis of the overall mechanism by which an electrochemical process between both metals improves HER performance. The new electrocatalyst developed in this work holds promise for membrane water electrolyzers.

## 2. Results and Discussion

### 2.1. Structural, Surface, and Morphological Properties

**Figure 1:**
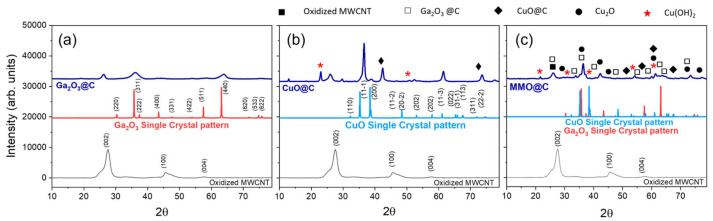
Powder X-ray diffraction (PXRD) patterns of (**a**) Ga2O3@C (**b**) CuO@C, and (**c**) MMO@C.

The XRD powder patterns of Ga_2_O_3_@C and CuO@C pure composites are shown in Figure 1a,b, respectively. For better visualization, the oxidized MWCNTs, Ga_2_O_3,_ and CuO single crystal diffractograms were included. For the oxidized MWNCTs, the characteristic diffraction peaks at 2θ 26.05° and 43.03° correspond to the planes (002) and (100), indicating multi-layered carbon nanotubes with concentric overlapping graphene sheets. In Figure 1a, the appearance of only three diffraction peaks is seen for Ga_2_O_3_@C, attributed to the (002) plane of the MWCNTs and cubic Ga_2_O_3_ (Fd-3m ICSD- 194506) [32], whereas in Figure 1b for CuO@C a large number of diffraction peaks are seen. Therefore, in the so-called CuO@C pure composite the presence of monoclinic CuO (C2/c ICSD- 1011148) [33] as well as some impurities of Cu(OH)_2_ and Cu_2_O are detected. Consequently, the XRD characterization indicates that Ga_2_O_3_@C is structurally more homogeneous than CuO@C, and in both materials disappearance of the plane (100) of MWNCTs at 2θ 43° is detected. This is an important finding due to the disappearance of the (100) plane in MWCNTs, with the composites indicating a modification of the interlayer or collapse of the structure [34]. Zhu et al. report that the strong interactions between cobalt oxide and the carbon framework in Co-bpdc/MWCNT composites disrupt the graphitic structure with the disappearance of the (100) plane after calcination [35]. Abdullah et al. report significant changes in the intensity and position in the XRD patterns of MWCNTs decorated with iron oxide layers [36], attributing the composite structure of metal oxide layers shielding the underlying carbon structure with the disappearance of the (100) diffraction peaks. Oke et al. highlight that an electronic or structural change in MWCNTs decorated with some metal oxide produces a reconfiguration of the carbon network which is responsible for the disappearance of certain peaks [37]. For that reason, the disappearance of the (100) plane in Ga_2_O_3_@C and CuO@C indicates that the MWCNTs were successfully decorated by the gallium and copper oxides, altering the interlayer spacing and disrupting the graphitic structure [34,35,36,37]. Regarding the XRD pattern of MMO@C (Figure 1c), it shows several diffraction peaks indicating planes of different oxide components present which are decorating the MWCNTs [38,39,40]. A decrease in the 2θ 25° peak indexed to the (002) plane of the hexagonal graphite structure of the MWCNTs is also observed along with well-defined diffraction peaks belonging to Ga_2_O_3_ and CuO, and Cu(OH)_2_ and Cu_2_O, as segregated phases [41,42]. The small number of sharp peaks in Figure 1c also indicates the presence of heterogeneous amorphous species, which could play significant roles in determining the final characteristics of these materials and are essential for their application in various fields [43]. The functional groups present in carbon nanotubes (such as carboxyl groups (COOH)) interact with the surface of the oxide nanoparticles, generating interactions that modify the electronic configuration and the arrangement of the atoms in the surface layers of the oxide, altering its crystalline network or deformation (strain) in the crystal lattice of the oxide. This deformation results in a change in the interplanar distances, shifting the peaks and making them wider and less intense.

**Figure 2:**
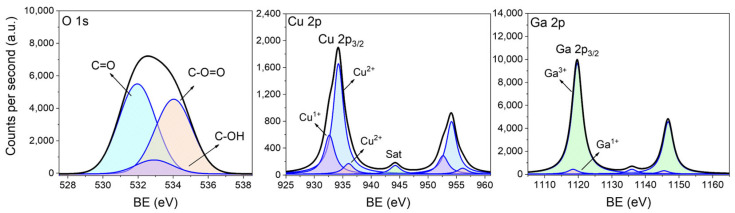
XP spectra of O1s, Cu 2p_3/2_, and Ga 2p_3/2_ for the MMO@C electrocatalyst.

Since the MMO@C electrocatalyst has several mixed oxides, XPS analyses of C 1s, O 1s, Ga 2p, and Cu 2p were carried out to investigate their surface chemical compositions. The binding energies for each element are summarized in Table 1. Surface C 1s (Figure S1) corresponds to the oxygenated functional groups and the O1s, Ga 2p, and Cu 2p spectra shown in Figure 2. The O 1s split into three peaks using the combination of Gaussian and Lorentzian deconvolution, indicating surface oxygen species at 532.0 eV (57%), 532.9 eV (6%), and 534.0 eV (37%) associated with oxygen bonds with different valence states. The Ga 2p_3/2_ energy level is almost a single peak that can be split into two surface species at 1118.7 eV (6%) and 1119.6 (94%). Similarly to O 1s analysis, no signal of Ga^3+^ associated with the CuGa_2_O_4_ spinel structure at 1117.3 eV is observed [44], indicating surface Ga_2_O_3_. The respective Cu 2p_3/2_ spectra indicate that Cu is present as a mixture of surface Cu^2+^ and Cu^+^ species. The three deconvolute peaks of Cu 2p_3/2_ spectra indicate surface Cu^+^ at 932.7 eV (33%) and Cu^2+^ as CuO at 934.2 eV (61%) and Cu(OH)_2_ at 936.0 eV (6%). The surface Ga/Cu atomic ratio calculated by comparing the corrected area under the curves (Table 2) was 6, which was larger than the nominal 2 for the formation of CuGa_2_O_4_ spinel. The Ga/Cu atomic ratio for MMO@C determined by EDX is 7.1 (Figure S2, Table S1), close to the surface Ga/Cu atomic ratio of 6 determined by XPS. The small difference obtained using both techniques is mainly because EDX is not a surface-sensitive technique, and its ratios are intermediate between the bulk and surface ratios [45]. Therefore, the surface enrichment of Ga and the presence of @C inhibits the formation of the spinel structure (CuGa_2_O_4_). Given the above, the proposed CuGa_2_O_4_@C corresponds to a mix of surface Ga_2_O_3_-CuO-Cu_2_O-Cu(OH)_2_@C and for the sake of clarity will be labeled as MMO@C.

**Figure 3:**
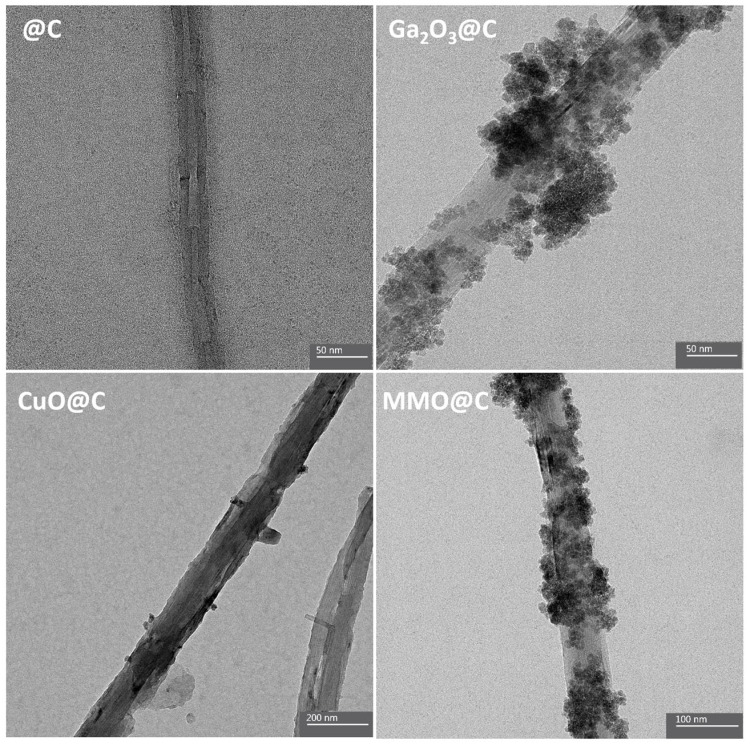
TEM images for @C, CuO@C, Ga2O3@C, and MMO@C electrocatalysts.

**Figure 4:**
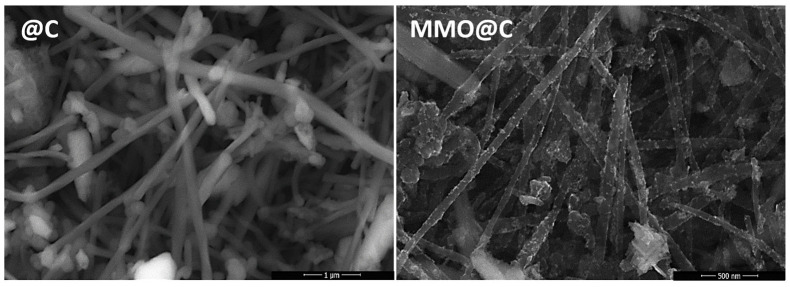
FESEM images for @C and MMO@C electrocatalysts.

The TEM images in Figure 3 provide morphological information about the surface of the synthesized materials. The elongated cylindrical structure characteristic of undecorated MWCNTs (@C) and morphological differences for the decorated systems can be noted. The Ga_2_O_3_@C electrocatalyst shows particle agglomerations covering a large part of the MWCNT surface, in contrast to the CuO@C electrocatalyst which presents isolated particles covering a considerably smaller portion of the MWCNT surface. Like the Ga_2_O_3_@C system, the MMO@C electrocatalyst exhibits similar particle agglomerations, indicating that the surface of the MMO@C is predominantly decorated with Ga_2_O_3_. This information is consistent with the surface atomic percentages measured by XPS, which reveal a surface enrichment of Ga with a Ga/Cu ratio equal to 6 (Table 2). The FESEM images in Figure 4 compare the morphology of the @C as blank and the MMO@C electrocatalyst. Similarly to the TEM analysis, the MMO@C electrocatalyst shows well-distributed agglomerations on the MWCNTs. The agglomerations have an average size of 8 nm, implying the formation of nanoparticles that decorate the surface of the MWCNTs. These nanoparticles correspond to the mixed metal oxides with no formation of spinel structure, according to X-ray (Figure 1) and XPS (Figure 2) analyses. Both the nanometer size of the particles and the surface distribution [46,47] added to their intrinsic electrochemical and physicochemical properties can influence their electrocatalytic performance towards the HER. Additionally, the BET surface area (S_BET_) and pore volume were measured for the @C, CuO@C, Ga_2_O_3_@C, and MMO@C electrocatalysts (Table 3). The N_2_ physisorption isotherms are shown in Figure S3, which shows a typical type II isotherm of non-porous materials. It is observed that the MMO@C electrocatalyst presents a larger S_BET_ (62 m^2^ g^−1^) compared to the Ga_2_O_3_@C (41 m^2^ g^−1^) and CuO@C (22 m^2^ g^−1^) electrocatalysts. Furthermore, the three decorated systems increased their S_BET_ compared to the @C undecorated system (13 m^2^ g^−1^). It is seen that the pore volume increases consistently with increasing S_BET_. The increase in the S_BET_ for the MMO@C electrocatalyst may be related to the formation of different surface species (Ga_2_O_3_; CuO; Cu_2_O and Cu(OH)_2_). The largest surface area for the MMO@C electrocatalyst allows a greater exposure of its available electroactive sites to improve the performance towards electrocatalytic reactions [48].

### 2.2. Electrochemical Characterization

**Figure 5:**
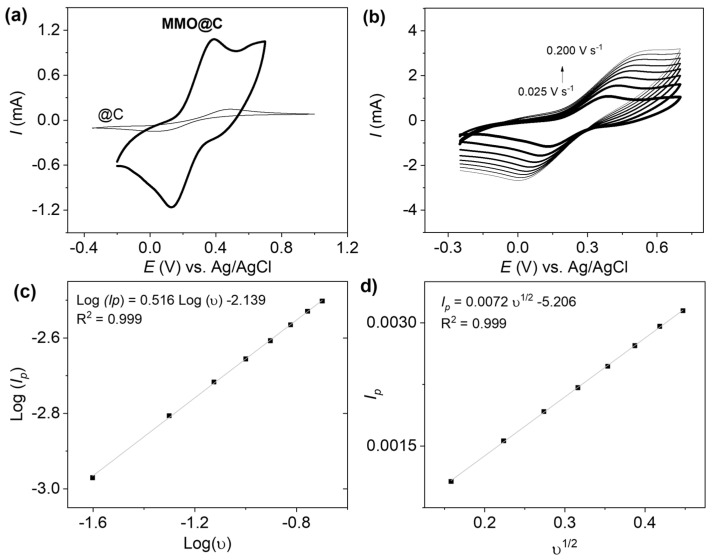
(**a**) Cyclic voltammograms for @C and MMO@C electrocatalysts, (**b**) scan rate study (0.025; 0.050; 0.075; 0.100; 0.125; 0.150; 0.175; and 0.200 V s^−1^), (**c**) plot Log (*I_p_*) vs. Log (υ) obtained from Figure 5b, and (**d**) plot *I_p_* vs. υ^1/2^ obtained from Figure 5b for MMO@C electrocatalyst towards a 0.1 M ferri/ferrocyanide redox couple, N_2_ sat.

Figure 5a shows the cyclic voltammograms for the @C and MMO@C electrocatalysts towards a ferri/ferrocyanide redox couple, where it is observed that ∆*E_p_* values are 252 mV for MMO@C and 465 mV for @C. Thus, both systems are electrochemically quasi-reversible, and due to the lower ∆*E_p_* value MMO@C has a higher electron transfer rate than @C [49]. Likewise, MMO@C presents a peak current approximately 10 times higher compared to @C, which indicates that mixed metal decoration on CNTs considerably improves the electrochemical properties of the electrocatalyst. In this way, MMO@C has the capacity to convert a greater amount of electroactive species [50]. Figure 5b shows the scan rate study of the MMO@C electrocatalyst towards a ferri/ferrocyanide redox couple between 0.025 and 0.200 V s^−1^. With the values of peak current (*I_p_*) and scan rate (*υ*) (Figure 5b), the *Log (I_p_)* vs. *Log (υ)* graph in Figure 5c is obtained, and the line equation shows a slope of 0.516. Since the slope is very close to 0.5, the system is diffusion-controlled [51]. Then, the slope obtained in Figure 5d corresponding to the linear equation *I_p_* vs. *υ*^½^ is 0.0072 is replaced in the Randles–Sevcik equation for reversible systems controlled by diffusion (Equation (1)), which is as follows:

(1) $$\frac{I_{p}}{\upsilon^{1/2}}=\;{2.69\times 10}^{5}\;n^{3/2}\;C_{o}\;D^{1/2}\;A$$

where the number of electrons transferred (*n*) is 1; *C_o_* is the concentration of the electroactive species (1 × 10^−4^ mol cm^−3^); *D* is the diffusion coefficient, which is 6.5 × 10^−6^ cm^2^ s^−1^ for the ferrocyanide ion [52]; and *A* is the electroactive area to be determined. Replacing values, the MMO@C paste electrode has an electroactive area of 0.105 cm^2^, being around 3.4 times larger than its geometric area (0.031 cm^2^). The above implies a great availability of active sites to carry out electrochemical processes [53].

### 2.3. Electrocatalytic Study of the HER

**Figure 6:**
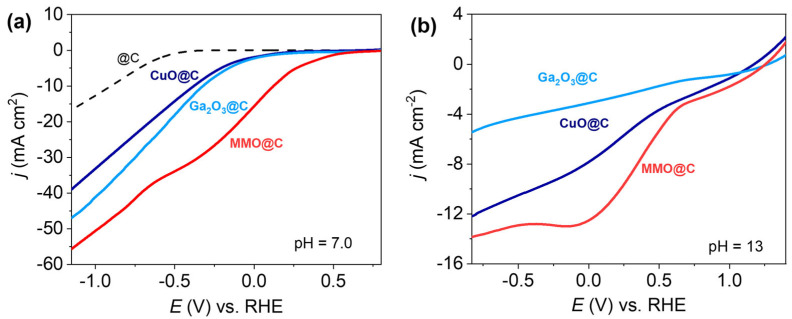
(**a**) Linear voltammograms in PBS buffer pH = 7.0; (**b**) linear voltammograms in 0.1 M KOH, N_2_ sat., and υ = 0.01 V s^−1^.

Figure 6a shows the linear voltammograms towards the HER in PBS buffer at neutral pH for the MMO@C, Ga_2_O_3_@C, CuO@C, and @C paste electrodes. The electrocatalytic performance summarized in Table 3 indicates that a large improvement in terms of onset potential (*E_o_*) was obtained for all three decorated systems compared to @C, and differences in their electrocatalytic behavior is clearly observed. Particularly, MMO@C (*E_o_* = 0.12 V vs. RHE) presents an outstanding improvement compared to its respective simple metal oxides Ga_2_O_3_@C (*E_o_* = −0.33 V vs. RHE) and CuO@C (*E_o_* = −0.38 V vs. RHE). Regarding MMO@C, its *E_o_* value is 450 mV and 500 mV higher than Ga_2_O_4_@C and CuO@C, respectively, proving to be energetically more favorable for initiating the HER. The large synergistic effect for the MMO@C electrocatalyst is not only expressed through its remarkable *E*_o_ value but also reaches a higher current when the potential magnitude is high (E ~ −1 V) (Figure 6a). It can be observed that the MMO@C electrocatalyst at the onset of the current drop associated with the HER exhibits a parallel cathodic process centered at ~ −0.25 V. To understand the cause of this cathodic process, the electrocatalytic performances of the MMO@C, Ga_2_O_3_@C, and CuO@C electrocatalysts were studied in 0.1 M KOH solution (Figure 6b). Analogously to what occurs in a neutral medium (Figure 6a), the synergistic effect between Cu and Ga in MMO@C is maintained under alkaline conditions for both current and potential. However, a great difference is evident for the individual metal oxides in alkaline medium, obtaining an electrocatalytic improvement of CuO@C over Ga_2_O_3_@C. Additionally, in Figure 6b the CuO@C and MMO@C paste electrodes show a cathodic process centered at ~ 0 V vs. RHE, with a more pronounced reduction process for MMO@C. This result indicates that the presence of Ga in MMO@C favors a potential shift towards positive values, facilitating the anodic process associated with the presence of Cu. This anodic process is also observed at neutral pH for the MMO@C electrocatalyst centered at ~ −0.25 V, but not for CuO@C (Figure 6a). According to the Pourbaix diagram for copper [54], when a potential sweep is applied towards negative values in neutral and alkaline conditions there is a tendency for Cu(I) species to form Cu(s). The XRD results (Figure 1) indicate that the MMO@C electrocatalyst corresponds to a mixture of metals oxides. The Ga/Cu surface atomic ratio of 6 for MMO@C obtained by XPS indicates a surface enrichment Ga and confirms the XRD results about the presence of segregated phases. The surface composition of the MMO@C electrode demonstrates that despite its lower abundance CuO can generate a notable electrocatalytic effect towards the HER (Figure 6a), helped by the presence of Ga_2_O_3_. The higher standard reduction potential value of the Cu^2+^/Cu^+^ pair (E° = +0.15 V) compared to the Ga^3+^/Ga^2+^ pair (E° = −0.65 V) indicates that when both metals are together gallium has a large tendency to remain in its oxidized form, donating electron density. Since the resonant system of the MWCNTs promotes the movement of electrons through their structure [28], the donation of electron density between both metals could also be favored by the conjugated π system of the MWCNTs, facilitating the electrochemical reduction of Cu^2+^ to Cu^+^ to produce the Cu(I) species on the electrode. According to the Pourbaix diagram [54], these potential and pH conditions produce Cu(s). The above is evident through the cathodic process in an alkaline medium centered at ~ 0 V, corresponding to Cu^2+^ + e^−^ → Cu^+^ (Figure 6b). In this way, the electrochemical reduction to produce Cu(I) facilitated by the electronic donation of Ga^3+^, in neutral and alkaline conditions, drives the generation of Cu(s) active sites on the MWCNTs, reducing the requirement for proton adsorption on the electrocatalyst [55].

**Figure 7:**
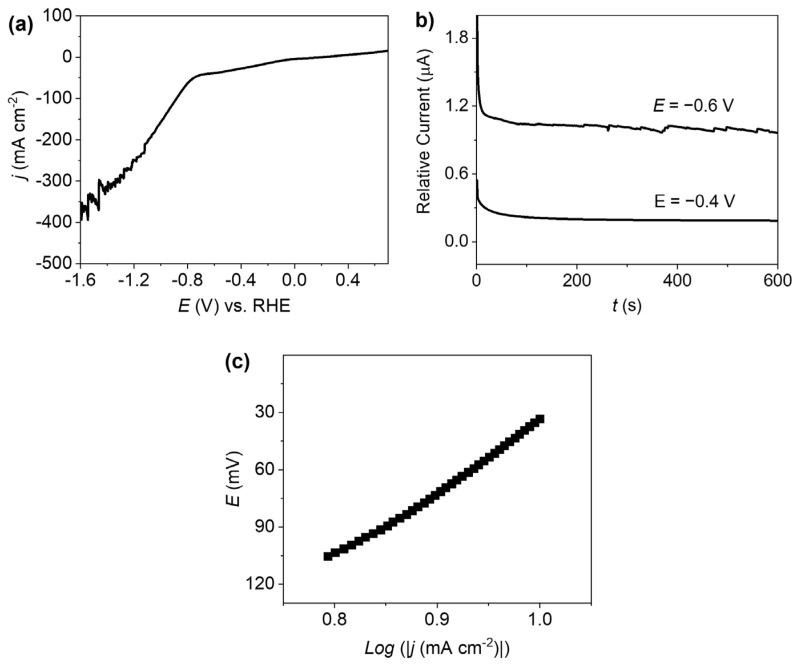
(**a**) Linear voltammogram towards the HER in 0.5 M H2SO4 using the MMO@C electrocatalyst. (**b**) Chronoamperometric study in 0.5 M H2SO4 using the MMO@C electrocatalyst at E = −0.4 and −0.6 V. (**c**) The Tafel slope (350 mV dec−1) for the MMO@C electrocatalyst.

A chronoamperometric study was performed for the MMO@C electrocatalyst as an electrolysis test to identify if the HER occurs during the cathodic process associated with CuO reduction. The voltametric profile in acidic conditions (0.5 M H_2_SO_4_) makes it easier to visualize the production of H_2_ bubbles on the electrode [56,57], as shown in Figure 7a. As expected, in acidic conditions higher magnitudes of currents are observed when applying a potential sweep towards negative values in an acid solution, indicating a greater H_2_ production than in neutral or alkaline conditions. The chronoamperometry performed at a fixed potential of −0.4 V and −0.6 V vs. RHE on the surface of MMO@C is presented in Figure 7b. At a fixed potential of −0.6 V, the characteristic noise associated with the formation of gaseous species identified as H_2_ bubbles is observed. This result verified that the HER occurs during the reduction process prior to ~−0.8 V (Figure 7a), where there is an inflection point and hydrogen production becomes more evident. Additionally, the open circuit potential (OCP) of the MMO@C electrocatalyst at pH = 7.0 is 0.72 V vs. RHE (Figure S4). When two metals are together in the same solution, the OCP corresponds to the highest possible potential difference without applying an external potential [58]. Therefore, the OCP defines the capacity of the electrode to oxidize or reduce [59]. In this sense, the electrochemical interaction of the different species in the MMO@C system tends to occur at a potential of 0.72 V, relatively far from the onset potential in a neutral medium (Figure 6a).

The Tafel slope was determined in an acidic medium to decrease the contribution of CuO reduction in the global reaction mechanism. In general terms, the Tafel slope represents the intrinsic activity of electrocatalysts toward the HER, and smaller Tafel slopes are related to faster HER rates [60]. Three possible reaction steps are usually accepted for the HER associated with the obtained Tafel slope values [60]. The Volmer steps in acid and alkaline medium (Equation (2)) correspond to the formation of hydrogen adsorbed on the electrocatalyst (MH_ads_) through a process known as proton discharge. The Heyrovsky steps (Equation (3)) correspond to H_ads_ binding to a proton or water in an alkaline medium, and through the contribution of electrons provided by the electrocatalyst gaseous H_2_ is generated. The Tafel steps in acid and alkaline medium (Equation (4)) are where two adsorbed hydrogens on nearby active sites (2MH) combine to give rise to H_2_. Considering that the Heyrovsky and Tafel correspond to electrochemical desorption reactions, it is proposed that H_2_ must be produced by a combination of Volmer with Heyrovsky or Tafel steps. If the Volmer step is rate limiting, the Tafel slope is close to 120 mV dec^−1^ and indicates a mechanism associated with slow kinetics. Whereas, if the Heyrovsky step is the rate-limiting reaction then the Tafel slopes must be closer to 40 mV dec^−1^, and if the process is limited by the Tafel step then the expected slope values are 30 mV dec^−1^ [61]. Even though the Tafel slope values guide the identification of HER mechanisms, it should be noted that these calculations are based on a set of assumptions that are not universally accepted as a clear identification of HER mechanisms [61,62]. According to the literature, it has been studied that some Ni/Zn and Ni/Al pressed powder electrodes have shown Tafel slopes over 120 mV dec^−1^, even reaching 307 mV dec^−1^ towards the HER, where the high Tafel slopes are related to a larger apparent surface area [63]. Additionally, it has been reported that some carbon paste electrodes have Tafel slopes between 186 and 508 mV dec^−1^ [64]. In accordance with the above, a Tafel slope of 240 mV dec^−1^ for a porous carbon electrode was found [65]. Within this context it has been studied that an excessive thickness of the catalyst layer influences the resistance to mass transport of H_2_ through the catalyst layer [62,66], resulting in high Tafel slopes, which is difficult to modulate when porous paste electrodes with a large surface area are used. However, it is known that a large surface area value, as in the case of CNT-based materials, can lead to a better electrocatalytic effect [29,67] and to obtaining higher faradaic currents [68] given the exposure of active sites available for the electrochemical process to occur.

In this work, the Tafel slope of the MMO@C electrocatalyst is 350 mV dec^−1^, which is higher than 120 mV dec^−1^. A high value of electroactive area was previously calculated for the MMO@C electrocatalyst (0.105 cm^2^, about 3.4 times larger than its geometric area), and this high value is associated with the high porosity and surface area of the decorated CNTs. On the one hand, as previously discussed, the large electrode surface may be related to a high value of the Tafel slope [62,63,66]. On the other hand, although the Tafel slope was obtained in an acidic medium, two reactions are occurring in parallel in the study area making it difficult to establish an HER mechanism associated only with the Tafel slope. Although the Tafel slope reveals slow global kinetics, the MMO@C electrocatalyst presents an excellent initiation potential in neutral medium (Figure 7a), being a good candidate to be coupled to renewable energy sources to produce green hydrogen.

(2) $$\begin{array}{c} \mathrm{Volmer}\;\mathrm{step}\;\mathrm{in}\;\mathrm{acid}\;\mathrm{medium}:\;H^{+}+M+e\;⇌\;{MH}_{ads}.\\ \mathrm{Volmer}\;\mathrm{step}\;\mathrm{in}\;\mathrm{alkaline}\;\mathrm{medium}:\;H_{2}O+M+e\;⇌\;{MH}_{ads}+{OH}^{-}. \end{array}$$

(3) $$\begin{array}{c} \mathrm{Heyrovsky}\;\mathrm{step}\;\mathrm{in}\;\mathrm{acid}\;\mathrm{medium}:\;MH+H^{+}+e\;⇌\;{M+H}_{2}.\\ \mathrm{Heyrovsky}\;\mathrm{step}\;\mathrm{in}\;\mathrm{alkaline}\;\mathrm{medium}:\;MH+H_{2}O+e\;⇌\;M+{OH}^{-}{+H}_{2}. \end{array}$$

(4) $$\mathrm{Tafel}\;\mathrm{step}\;\mathrm{in}\;\mathrm{acid}\;\mathrm{and}\;\mathrm{alkaline}\;\mathrm{medium}:\;2MH\;⇌\;2{M+H}_{2}.$$

### 2.4. Quantitative Study

To quantify the H_2_ production monitored by GC, the electrolysis was performed at a fixed potential of −0.8 V vs. RHE for 3 h using an MMO@C paste electrode in 0.5 M H_2_SO_4_ (pH = 0.3). Once again, an acidic solution was used, since having a higher concentration of H^+^ in the medium favors a better quantification of H_2_ gas. The plot of charge vs. time during the electrolysis (Figure S5) shows the progressive increase in charge magnitude on the electrode surface throughout the electrolysis, reaching a final charge of 83.9 C. The total volume of the sealed electrochemical cell is 30 mL; therefore 26 mL of solution was used, leaving a head space of 4 mL for the accumulation of the gaseous product. After 3 h, 50 μL of gas was extracted from the headspace to be injected into the GC to obtain a chromatographic area of 1,083,696 μV/s. Then, to calculate the amount of hydrogen produced during electrolysis, a calibration curve obtained in a previous work was used [69]. It was determined that the MMO@C electrode produces a large amount of 4.1 × 10^−4^ mol of H_2_, corresponding to 1.31 × 10^−2^ mol cm^−2^. The charge on the electrode surface at *E_o_* is calculated from the ratio between faradaic current and time (Equation (5)), and time is obtained from Equation (6). These are as follows:

(5) Q=It

(6) $$t=\frac{Eo}{\upsilon }$$

where *Q* is the charge on the electrode surface at *E_o_* (C), *I* is the Faradaic current at *E_o_* (1.6 × 10^−4^ A), and *t* is the time at *E_o_*. Given that *E_o_* in an acidic medium is −0.13 V and the scan rate (υ) is 0.01 Vs^−1^, it is calculated that *t* = 13 s and therefore *Q* = 0.002 C. The number of active sites is calculated from Faraday’s Law of Electrolysis (Equation (7)), which is as follows:

(7) $$N=\frac{Q}{nF}$$

where *N* is the number of active sites, *Q* is the charge on the electrode surface at *E_o_* previously calculated (0.002 C), *F* is the Faraday constant (96,485 C∙mol^−1^), and *n* is the number of electrons transferred (2 for HER). Thus, *N* = 1.04 × 10^−8^ mol. The turnover number (TON) measures the total activity of a catalyst and represents the total number of substrate molecules converted by a single catalyst molecule before it becomes inactive. The turnover frequency (TOF) measures the catalyst’s instantaneous efficiency and indicates the number of catalytic cycles, or molecules converted per active site per unit time. The TON and TOF for MMO@C electrocatalyst are obtained from the following Equations (8) and (9), respectively:

(8) $$TON=\frac{n_{H_{2}}}{N}$$

(9) $$TOF=\frac{TON}{t}$$

where $n_{H_{2}}$ is the number of moles of hydrogen produced by the MMO@C electrocatalyst (4.1 × 10^−4^ moles), *N* = 1.04 × 10^−8^ mol, and *t* is electrolysis time (3 h). Replacing values, it is determined that TON = 39,423 and TOF = 13,141 h^−1^. Finally, faradaic efficiency *FE* (%) is obtained through Equation (10), which is as follows:

(10) $${FE}_{H_{2}}\left(\%\right)=\frac{\;n_{H_{2}}n\;F}{q}\;100$$

Since the amount of hydrogen produced ($n_{H_{2}}$) is 4.1 × 10^−4^ mol, the number of electrons transferred for the HER (*n*) is 2, the Faraday constant (*F*) is 96,485 C∙mol^−1^, and the final charge (*q*) after electrolysis is 83.9 C (Figure S5), so *FE* (%) is 94.3%.

## 3. Materials and Methods

### 3.1. Chemicals

Multi-walled carbon nanotubes (MWCNTs) powder, sodium hydroxide (pellets for analysis), sodium chloride, and potassium chloride were purchased from Merck, Chile. Potassium ferricyanide (K_3_[Fe(CN)_6_]) 98.5%, potassium ferrocyanide (K_4_[Fe(CN)_6_]), potassium hydroxide, potassium dihydrogen phosphate 99.5%, disodium hydrogen phosphate 99%, sulfuric acid 98%, absolute ethanol, copper (II) nitrate, and gallium (III) acetylacetonate were obtained from Sigma-Aldrich, Chile. Ultra-pure water was provided by Millipore-Q system (18.2 MΩcm). All salts are of analytical grade.

### 3.2. Synthesis of Electrocatalysts

The chemical oxidation of MWCNTs was carried out using 1 g of MWCNTs sonicated in 150 mL of a 3:1 (*v*/*v*) H_2_SO_4_/HNO_3_ mixture for 6 h at 70 °C under a fume hood. The mixture was allowed to cool to room temperature, then filtered and washed with ultra-pure water until neutralization was complete (pH = 7). The obtained solid product was dried in an oven at 105 °C for 5 h. The synthesis of electrocatalysts was carried out by mixing 0.3 g of previously oxidized MWCNTs with 4.124 mmol of copper nitrate for CuO@C and 4.124 mmol of gallium acetylacetonate for Ga_2_O_3_@C, respectively. The mixtures were homogenized in an agate mortar and then sonicated for 20 min in ethanol. After evaporation of ethanol, the obtained solid was added to 35 mL of 0.05 M NaOH in a Teflon-lined stainless steel autoclave apparatus for hydrothermal treatment at 180 °C for 12 h. The resulting solid was filtered, washed with ultra-pure water (Milli Q), and finally dried in an oven at 105 °C for 5 h. For MMO@C synthesis, the same procedure described above was performed using 1.375 mmol of copper salt and 2.750 mmol of gallium salt. The characterization results indicate the presence of a mixture of metal oxides (MMOs) without a spinel structure. Therefore, the materials were labeled as CuO@C, Ga_2_O_3_@C, and MMO@C.

### 3.3. Paste Electrodes Preparation

The paste electrodes contain a mixture of decorated MWCNTs and mineral oil in a 7:3 (*w*/*w*) ratio. The paste electrodes were prepared with a 7:3 proportion (*w*/*w*) of the MWCNTs-decorated material (CuO@C, Ga_2_O_3_@C, and MMO@C) and mineral oil, respectively. The mixture was homogenized in an agar mortar, adding diethyl ether. Also, a blank electrode was prepared only with undecorated MWCNTs following the above methodology. When diethyl ether was completely evaporated, the pastes were packed into the Teflon hollow electrodes and then heated in oven at 90 °C for 5 h. Finally, electrodes were allowed to cool at room temperature, and the surfaces were polished to a smooth finish. All the measurements with the paste electrodes were reproducible after surface renewing, showing the same voltametric profile and a current change below 3% for each measurement. The geometric area of all the electrodes was 0.031 cm^2^.

### 3.4. Instrumentation

The electrocatalytic activity of electrodes was studied by using a three-compartment electrochemical cell. One compartment is available for the reference electrode Ag/AgCl (3 M KCl), a second compartment for the counter electrode (Pt spiral of great area), and a third compartment for the working electrode, which is separated from the counter electrode compartment by a porous medium. The transformation between Ag/AgCl and RHE reference electrodes is *E_RHE_* = *E_Ag/AgCl_* + 0.059pH + *E^0^_Ag/AgCl_*; *E^0^_Ag/AgCl_* = 0.1976 V. Linear and cyclic voltametric analyses and electrolysis were performed with a CH Instruments 650E potentiostat. Electrolysis for H_2_ quantification was performed at −0.8 V vs. RHE fixed potential for the HER. Powder X-ray diffraction (PXRD) patterns were collected at room temperature on a Bruker D8 Advance diffractometer (Bruker, Billerica, MA, USA) equipped with a Cu Kα radiation source, in a range of 5° < 2θ < 80. For the morphological study, the electrocatalysts were analyzed by transmission electron microscopy (TEM) Talos F200X G2 (Thermo Fisher Scientific, Hillsboro, OR, USA) and field emission scanning electron microscopy (FE-SEM) with energy dispersive X-ray spectroscopy (EDX) QUANTA FEG250 (Thermo Fisher Scientific, Hillsboro, OR, USA). X-ray photoelectron spectroscopy (XPS) of the electrocatalysts was recorded on a VG Escalab 200R electron spectrometer (Thermo Fisher Scientific, Waltham, MA, USA) using a Mg Kα (1253.6 eV) photon source. The binding energies (BEs) were referenced to the C 1s level of the carbon support at 284.8 eV. An estimated error of ± 0.1 eV can be assumed for all measurements. The intensities of the peaks were calculated from the respective peak areas after background subtraction and spectrum fitting by standard computer-based statistical analysis, which included fitting the experimental spectra. Nitrogen physisorption isotherms of the electrocatalysts were obtained at −196 °C using a Micromeritics TriStar II 3020 instrument (Micromeritics Instrument Corporation, Norcross, GA, USA) to evaluate the BET specific surface area (S_BET_). Prior to the measurements, the samples were degassed under vacuum at 200 °C for 3 h in a nitrogen flow. S_BET_ was calculated from the adsorption branch in the range 0.05 ≤ P/Po ≤ 0.25. Hydrogen production was monitored using a completely sealed two compartment electrolysis cell for the most electrocatalytic system (MMO@C paste electrode). During the 3 h of electrolysis, the 0.5 M Ar-saturated sulfuric acid solution (pH = 0.3) was kept in agitation with a magnetic stirring bar, aiming to favor hydrogen bubbles spread to the head space. During the experiment, 50 μL of gas was extracted from the head space and analyzed by gas chromatography (GC-2030 Plus, Shimadzu, Japan) with a thermal conductivity detector (TCD) Subsequently, a correction was applied considering the total volume of the head space (4 mL). The GC operated under the following working conditions: injector temperature = 50 °C; P = 248 kPa; column temperature = 25 °C; flow = 5.5 mL/min; carrier gas = Ar; and detector temperature = 200 °C.

## 4. Conclusions

Novel paste electrodes based on MWCNTs decorated with pure single oxides (Ga and Cu) and mixed metal oxides (Ga with Cu) were synthesized to be used in the electrocatalytic reaction of the HER. Significant differences in the structural and surface properties of the MMO@C paste electrode relative to the pure electrodes were found. The excellent electrocatalytic performance of the MMO@C paste electrode is associated with the synergistic effect between gallium and copper oxides on the electrode surface and the formation of surface mixed species of Ga_2_O_3_-CuO-Cu_2_O-Cu(OH)_2_@C. It is worth noting that although the Tafel slope reveals slow reaction kinetics, the H_2_ production of 1.31∙10^−2^ mol∙cm^−2^ with a TON of 39423, a TOF of 13,141 h^−1^, and a faradaic efficiency of 94.3% indicates the potential of the MMO@C electrocatalyst to produce green H_2_. Furthermore, its outstanding onset potential of +0.12 V vs. RHE in a neutral medium allows it to be coupled to renewable energy production systems.

## Figures and Tables

**Table 1:** Binding energies (eVs) and composition (%) for each energy level and species in the MMO@C electrocatalyst.

Energy Level	Species	Binding Energy (eV)	Composition (%)
C 1s	C-C	284.7	50
	C-O, C-OH	286.2	28
	C=O	287.6	6
	O-C=O	288.9	16
O 1s	C=O	532.0	57
	C-OH	532.9	6
	O-C=O	534.0	37
Ga 2p	Ga^1+^ (Ga_2_O)	1118.7	6
	Ga^3+^ (Ga_2_O_3_)	1119.6	94
Cu 2p	Cu^1+^ (Cu_2_O)	932.7	33
	Cu^2+^ (CuO)	934.2	61
	Cu^2+^ (Cu(OH)_2_)	936.0	6

**Table 2:** Atomic percentages of elements obtained by XP spectra for the MMO@C electrocatalyst.

C (%)	O (%)	Ga (%)	Cu (%)	Ga/Cu Atomic Ratio
60	26	12	2	6

**Table 3:** BET surface area (S_BET_), pore volume, and onset potential (*E_o_*) at −10 mA cm^−2^ towards the HER at pH = 7.0 for electrocatalysts.

Electrocatalyst	S_BET_ (m^2^ g^−1^)	V Pore (cm^3^ g^−1^)	*E_O_* (V) vs. RHE, pH = 7.0
@C	13	0.017	−0.90
CuO@C	22	0.047	−0.38
Ga_2_O_3_@C	41	0.066	−0.33
MMO@C	62	0.102	+0.12

## Data Availability

Data are contained within the article.

## Supplementary Materials

The following supporting information can be downloaded at: https://www.mdpi.com/article/10.3390/ijms26189057/s1.
