# Supplementary material for: New Paste Electrode Based on Copper and Gallium Mixed Metal Oxides-Decorated CNT for Highly Electrocatalyzed Hydrogen Evolution Reaction
Source: Int J Mol Sci. 2025 Sep 17;26(18):9057. doi: 10.3390/ijms26189057 (PMC12470142; doi:10.3390/ijms26189057)
Supplement: Supplementary file 1 [file ijms-26-09057-s001.zip › ijms-3825436-supplementary.pdf]

*Supplementary Materials*

# New Paste Electrode Based on Copper and Gallium Mixed Metal Oxides Decorated CNT for Highly Electrocatalyzed Hydrogen Evolution Reaction

**Claudio Barrientos, Silvana Moris, Dana Arias, Gina Pecchi, José Ibarra, Galo Ramírez * and Leyla Gidi ***

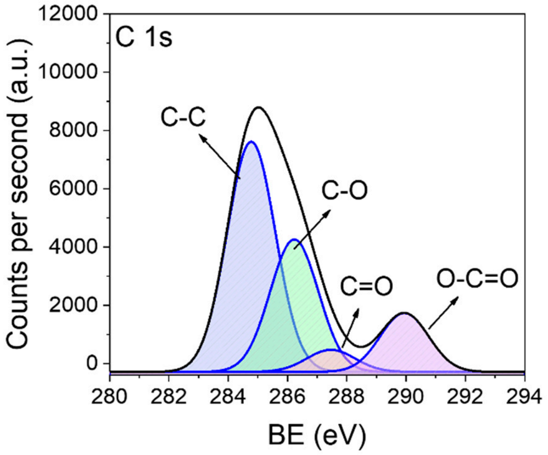


**Figure S1.** XP spectra of C 1s for MMO@C electrocatalyst.

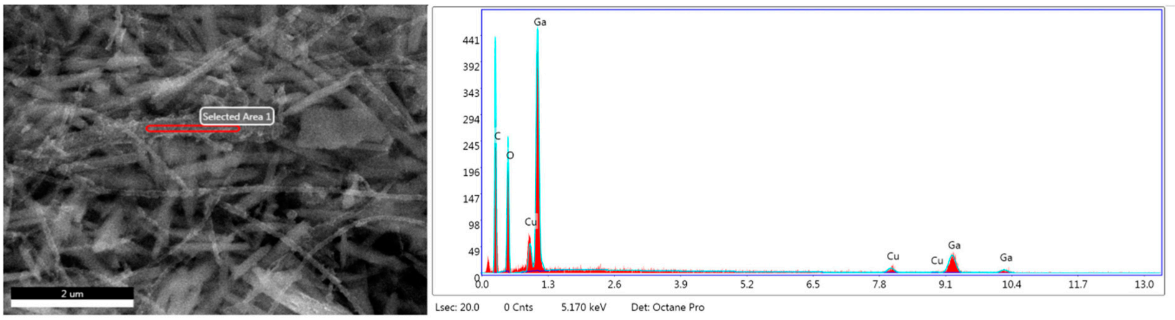


**Figure S2.** FESEM-EDX Analysis of MMO@C electrocatalyst.

**Table S1**. Atomic percentages of the elements detected by EDX analysis for the MMO@C electrocatalyst.

| Element | Atomic percentage (%) |
|---|---|
| C | 63.4 |
| O | 30.9 |
| Cu | 0.7 |
| Ga | 5.0 |

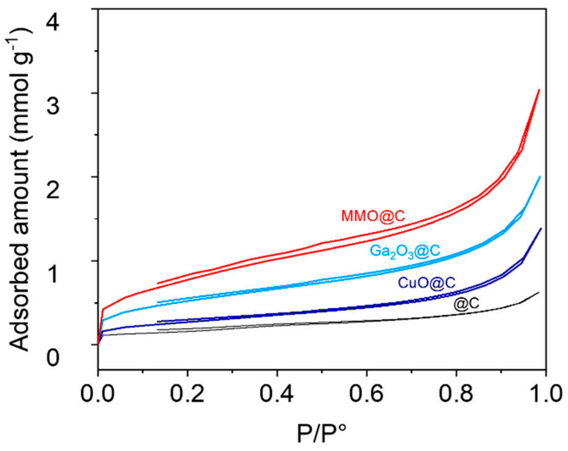


Figure S3. $N_2$ physisorption isotherms (surface area ($S_{BET}$)) for @C, CuO@C, Ga2O3@C and MMO@C electrocatalysts.

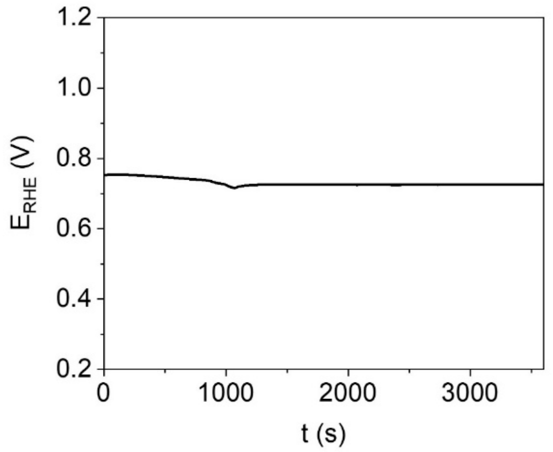


**Figure S4.** OCP for MMO@C electrocatalyst in PBS buffer pH = 7.0. The initial value of OCP is 0.75 V and during the first 1000 s it experiences a drop until reaching a minimum of 0.71 V. Then the value of OCP stabilizes, remaining at 0.72 V.

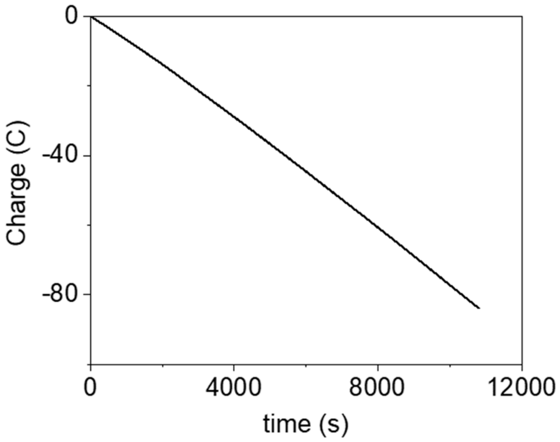


**Figure S5.** Plot Charge vs. time during electrolysis at $E$ = -1 V using MMO@C paste electrode in 0.5 M $H_2SO_4$, Ar sat. Final charge ($q$) = 83.9 C.
